# Development and external validation of a risk prediction model for early peripheral intravenous catheter failure in outpatients and emergency department patients receiving consecutive infusion therapy

**DOI:** 10.3389/fmed.2026.1963710

**Published:** 2026-10-08

**Authors:** Jingjue Mao, Caifeng Xu, Yuxin Yao, Yifan Jiang, Yangyang Li, Yan Peng, Sibei Wan, Mengru Liu, Congcong Cao, Yunqing Zhou

**Affiliations:** 1 Department of Nursing, Shanghai General Hospital, Shanghai, China; 2 Tongji University School of Medicine, Shanghai, China; 3 Guangzhong Road Community Health Service Center, Shanghai, China

**Keywords:** catheter failure, emergency department, external validation, nursing management, outpatient infusion, peripheral intravenous catheter, risk prediction model

## Abstract

**Background:**

Peripheral intravenous catheter (PIVC) failure is a common complication during intravenous infusion therapy and may result in treatment interruption, repeated venipuncture, and increased healthcare burden. For outpatients and emergency department patients receiving consecutive infusion therapy, early identification of patients at risk of PIVC failure before discharge with a catheter in place remains challenging. This study aimed to develop and externally validate a risk prediction model for early PIVC failure.

**Methods:**

This dual-center prospective cohort study included adult outpatients and emergency department patients receiving intravenous infusion therapy on consecutive days between January and June 2026. Patients from Shanghai General Hospital were included in the development cohort, and patients from Guangzhong Road Community Health Service Center, Hongkou District, Shanghai, were included in the external validation cohort. Eleven candidate predictors available during the initial visit and first infusion were incorporated into a multivariable logistic regression model. Model performance was assessed using discrimination, calibration, bootstrap validation, and decision curve analysis.

**Results:**

A total of 485 patients were included, including 340 in the development cohort and 145 in the external validation cohort. Early PIVC failure occurred in 112 (32.9%) and 48 (33.1%) patients, respectively. The number of special intravenous medication categories (OR=2.533, 95% CI: 1.755–3.657), serum albumin (per 5-g/L increase, OR=0.311, 95% CI: 0.221–0.436), and PIVC placement in the dominant limb (OR=4.718, 95% CI: 2.244–9.920) were significant predictors. The model showed good discrimination, with an apparent AUC of 0.862 in the development cohort, an optimism-corrected AUC of 0.840 after 1,000 bootstrap resamples, and an AUC of 0.845 in the external validation cohort. Decision curve analysis suggested potential clinical utility.

**Conclusions:**

This study developed and externally validated a risk prediction model for early PIVC failure among outpatients and emergency department patients receiving consecutive infusion therapy. The model may support early risk stratification, individualized catheter management, and clinical decision-making regarding PIVC retention after discharge.

## 1 Introduction

Peripheral intravenous catheters (PIVCs) are among the most commonly used vascular access devices in clinical practice because they are easy to insert, minimally invasive, and suitable for short-term intravenous therapy. For outpatients and emergency department patients requiring intravenous infusion therapy over several consecutive days, retaining the PIVC after the first infusion can reduce the need for repeated venipuncture and help maintain continuity of treatment. However, not all PIVCs remain functional until the completion of the planned treatment course. PIVC failure refers to the inability to continue using the original catheter before completion of the prescribed intravenous therapy because of phlebitis, infiltration or extravasation, catheter occlusion, catheter migration or dislodgement, or local infection, necessitating unplanned catheter removal or the establishment of new vascular access. A recent systematic review involving 478,586 PIVCs reported an all-cause failure rate of 36.4%, indicating that more than one-third of PIVCs failed before treatment completion (1). Another meta-analysis involving 76,977 catheters found that, among studies using clearly defined diagnostic criteria, the incidence rates of phlebitis, infiltration or extravasation, and catheter occlusion were 19.3%, 13.7%, and 8.0%, respectively (2). PIVC failure can result not only in localized pain, tissue injury, and treatment interruption but also in repeated venipuncture and additional healthcare costs, thereby increasing the burden on patients and the nursing workload.

PIVC failure results from the combined effects of multiple factors, including patient characteristics, venous conditions, catheter insertion site, and infusion therapy. Previous studies have shown that age, sex, venous conditions, insertion site, catheter placement in the dominant limb, and the infusion of irritant medications may be associated with PIVC failure (3, 4). During PIVC use, nurses should continuously assess the skin and vein at the insertion site, the integrity of catheter securement, and catheter patency, while closely monitoring for early signs of local complications such as infiltration, extravasation, and phlebitis (5). However, current clinical assessments focus primarily on catheter maintenance and the identification of abnormalities after insertion. Decisions regarding whether a patient is suitable for continued PIVC retention and discharge with the catheter in place after the initial infusion still rely largely on nurses’ clinical experience. Integrating demographic characteristics, venous conditions, treatment regimens, and laboratory indicators available at the initial visit and during the first infusion may enable individualized estimation of the short-term risk of PIVC failure, thereby shifting nursing management from reactive intervention after catheter failure to risk identification and decision support before patient discharge.

This issue is particularly relevant among outpatients and emergency department patients receiving infusion therapy on consecutive days. Unlike inpatients, these patients typically leave the healthcare facility after completing their daily infusion. Catheter care between infusions therefore depends largely on patients and their family members, while nurses are unable to continuously monitor catheter status. For patients scheduled to receive intravenous infusion therapy for three consecutive days, whether the initially inserted catheter remains functional through completion of treatment on day 3 directly determines whether PIVC retention achieves its intended purpose of reducing repeated venipuncture. Patients at low risk of short-term failure may avoid subsequent venipuncture by being discharged with the PIVC in place. In contrast, if a PIVC is routinely retained in a high-risk patient, occlusion, infiltration, or dislodgement may occur before the next visit or during the subsequent infusion. Such patients may still require new vascular access while also incurring the initial catheterization cost and experiencing inconvenience during physical activity, washing, and daily living while carrying the catheter. Therefore, the key objective of PIVC management in outpatient and emergency department settings is not simply to prolong catheter dwell time but to identify patients who are genuinely likely to benefit from discharge with the catheter in place. For high-risk patients, catheter protection, recognition of abnormal symptoms, and instructions regarding timely follow-up can be strengthened. For patients expected to derive limited benefit from short-term catheter retention, the necessity of retaining the PIVC and the suitability of alternative short-term vascular access options should be reassessed according to the planned infusion course and treatment characteristics.

Existing studies of PIVC failure have primarily involved hospitalized or critically ill patients and have largely focused on all-cause failure throughout the entire catheter dwell period or on individual complications such as phlebitis and infiltration (4, 6). Recent evidence from emergency department populations has also demonstrated that PIVC-related complications, including phlebitis and infiltration, remain clinically relevant in this setting, highlighting the importance of identifying factors associated with catheter complications and strengthening early catheter assessment and management (7). During PIVC dwell, hospitalized patients receive continuous observation and catheter care from healthcare professionals. Their activity environment, catheter protection conditions, and timing of abnormality detection differ substantially from those of outpatients and emergency department patients. Consequently, findings from inpatient studies cannot be directly applied to assessing the risk associated with discharge with a PIVC in place in outpatient and emergency department settings. For patients receiving infusion therapy for three consecutive days, maintaining catheter function from initial insertion through completion of treatment on day 3 has direct clinical relevance and decision-making value. However, studies predicting short-term PIVC failure using information available during the first infusion and externally validating the prediction model at an independent center remain limited in this specific clinical setting.

Accordingly, this dual-center prospective cohort study enrolled adult outpatients and emergency department patients with PIVCs who received intravenous infusion therapy on consecutive days. The cohort from the primary center served as the model development cohort. Demographic characteristics, disease-related factors, venous conditions, infusion therapy information, and laboratory indicators available at the initial visit and during the first infusion were used to develop a risk prediction model for early PIVC failure. A cohort from another independent center was used for external validation to evaluate the model's discrimination, calibration, and clinical utility. This study aimed to establish an early PIVC failure risk assessment tool for outpatients and emergency department patients receiving infusion therapy on consecutive days, providing a basis for nurses to assess the potential benefits of discharge with a PIVC in place, implement risk stratification and individualized health education, and further optimize short-term vascular access management.

## 2 Methods

### 2.1 Study design

This dual-center prospective cohort study comprised a model development cohort and an independent external validation cohort. Patients enrolled at Shanghai General Hospital constituted the model development cohort, which was used to develop and internally validate the risk prediction model for early PIVC failure. An independent group of patients concurrently enrolled at the Guangzhong Road Community Health Service Center, Hongkou District, served as the external validation cohort and was used to evaluate the model's predictive performance across different healthcare institutions within the same district. Both centers served a shared geographic catchment area, and patients in both study cohorts were drawn from Hongkou District, Shanghai. Both cohorts used identical eligibility criteria, variable definitions, data collection procedures, and outcome ascertainment criteria. The participants in the two cohorts were independent, with no overlap. The study was designed and reported in accordance with the Strengthening the Reporting of Observational Studies in Epidemiology (STROBE) statement and the Transparent Reporting of a Multivariable Prediction Model for Individual Prognosis or Diagnosis (TRIPOD) statement.

### 2.2 Study participants

Adult patients who received intravenous infusion therapy on consecutive days and underwent PIVC placement in the outpatient or emergency departments of the two study centers between January and June 2026 were consecutively enrolled. At Shanghai General Hospital, patients with more severe conditions were directed through the hospital triage process to the emergency department for acute assessment and treatment. Study enrollment was restricted to patients meeting the eligibility criteria below, including a planned three-day course of intravenous infusion therapy with PIVC retention after the first infusion. The hospital cohort therefore represented a specific infusion-treatment population and should not be regarded as representative of the overall emergency department population.

The inclusion criteria were as follows: (1) age ≥18 years; (2) scheduled to receive intravenous infusion therapy for three consecutive days, with the PIVC intended to remain in place after the first infusion for subsequent treatment; and (3) provision of written informed consent by the patient or a family member.

The exclusion criteria were as follows: (1) concurrent use of other vascular access devices, such as a midline catheter or central venous catheter; and (2) missing primary outcome data or key predictor variables that could not be retrieved or verified from the original medical records.

### 2.3 Sample size considerations

Eleven candidate predictors were prespecified, each contributing one regression parameter under the specified coding and linear functional forms. The sample size reflected consecutive enrollment of eligible patients during the study period. The development cohort included 340 patients, with 112 events and 228 non-events, corresponding to approximately 10.2 events per candidate predictor parameter, excluding the intercept. This ratio is a descriptive benchmark rather than definitive evidence of sample-size adequacy. Contemporary recommendations consider the outcome proportion, number of predictor parameters, anticipated model fit, and requirements for limiting overfitting and achieving sufficiently precise predictions.

To use the available development data efficiently, all 340 patients were used for model estimation, with 1,000 bootstrap resamples for internal validation rather than random data splitting. The independent external validation cohort included 145 patients, with 48 events and 97 non-events, and was not used for model estimation. Contemporary recommendations for external validation emphasize the precision of discrimination, calibration, and clinical utility estimates. Accordingly, this cohort provided an initial assessment of performance at an independent center, while its limited event count warrants caution, particularly when interpreting calibration and decision curve estimates.

### 2.4 Outcome definition

The primary outcome was early PIVC failure, defined as any PIVC failure occurring from successful initial catheter insertion and initiation of the first infusion to completion of the scheduled infusion on day 3. This observation period covered the principal treatment cycle for outpatients and emergency department patients receiving intravenous infusion therapy for three consecutive days.

PIVC failure was defined as the inability to continue using the original PIVC for subsequent infusions before completion of the planned intravenous therapy because of a catheter-related problem, necessitating unplanned catheter removal or the establishment of new vascular access. Catheter-related problems included catheter occlusion or inability to flush or infuse normally, infiltration or extravasation, phlebitis, catheter migration or dislodgement, and local infection. Patients who experienced any of these events at any time during the observation period, resulting in the inability to continue using the original PIVC, were classified as having early PIVC failure. Patients whose original PIVC remained functional through completion of the scheduled infusion on day 3 were classified as not having early PIVC failure.

Information on catheter-related symptoms or problems occurring at home between visits was obtained by questioning patients when they returned for the next day's infusion and through telephone interviews. Before each subsequent daily infusion, a nurse who had received standardized training assessed PIVC patency and the condition of the insertion site. Events were classified according to the prespecified definition of early PIVC failure by integrating the information reported by the patient with the nurse's assessment findings. Cases in which outcome classification remained uncertain were independently reviewed by a second vascular access specialist nurse, who determined the final outcome classification.

### 2.5 Predictor data collection

Eleven candidate predictors were prespecified on the basis of evidence from previous studies, clinical expertise, and the characteristics of PIVC management in outpatient and emergency department settings. All predictors were obtained at the initial visit or during the first infusion, ensuring that predictor assessment preceded the occurrence of the outcome.

The candidate predictors included age, sex, difficult intravenous access score, number of special intravenous medication categories, white blood cell count, serum albumin, C-reactive protein, hemoglobin, catheter placement in the dominant limb, catheter insertion on the dorsum of the hand, and history of diabetes mellitus (8).

Special intravenous medications were classified according to the predefined intravenous treatment regimen available before the first infusion. Based on formulation-related characteristics that could potentially increase peripheral venous irritation, these medications were categorized into three groups: (1) pH-related irritant medications, defined as medications with a pH, after preparation or under actual infusion conditions, that deviated from the physiological blood pH range and for which the prescribing information, pharmaceutical references, or previous studies suggested a potential for local venous irritation. Common examples in the present study included intravenous azithromycin, levofloxacin, aminophylline, pantoprazole, and omeprazole; (2) hyperosmolar medications, defined as medications that, under the actual clinical infusion regimen, imposed a relatively high osmotic load and could potentially increase peripheral venous irritation. Common examples included 20% mannitol, potassium chloride, calcium gluconate, calcium chloride, and high-dose intravenous vitamin C; and (3) particulate/excipient-related irritant medications, defined as medications that could potentially increase local venous irritation because of complex formulation characteristics, excipients or emulsifying systems, or a recognized risk of insoluble particulate formation, aggregation, or precipitation during preparation or administration. Common examples included compound multivitamin preparations for injection, Xuesaitong for injection, salvianolate for injection, Shenmai injection, and fat-soluble vitamin preparations for injection. Each medication category present in the predefined treatment regimen was assigned one point. The use of one or more medications within the same category was counted as one category, whereas different categories were counted separately. Accordingly, the number of special intravenous medication categories ranged from 0 to 3. The operational definitions of the three medication categories and representative medications included in each category are provided in Supplementary Table S1.

Laboratory indicators were obtained from tests performed on the same day before the first infusion and included white blood cell count, serum albumin, C-reactive protein, and hemoglobin. Continuous variables were retained in their original continuous form for analysis, whereas categorical variables were coded according to a prespecified data dictionary.

### 2.6 Data collection and quality control

Before study initiation, standardized data collection forms and a data dictionary were developed to define each predictor, its measurement method, timing of collection, and coding rules. Data collectors at both study centers received standardized training. Demographic and disease-related information was obtained from outpatient and emergency department medical records. The difficult intravenous access score, catheter insertion site, and dominant limb were assessed on site by trained nurses. Information on infused medications was obtained from the medication orders and administration records for the first infusion, while laboratory indicators were derived from test results obtained before the first infusion.

After initial PIVC insertion, patients were followed until completion of the scheduled infusion on day 3 or the occurrence of PIVC failure, whichever occurred first. Before each subsequent infusion, PIVC patency and the condition of the insertion site were assessed using standardized criteria. Following data entry, logical consistency checks were performed, and the data were verified against the original records. No candidate predictor or primary outcome data were missing in either the development cohort or the external validation cohort included in the final analysis.

### 2.7 Bias control

Eligible patients were consecutively enrolled at both study centers using identical inclusion and exclusion criteria to reduce selection bias. Before study initiation, the definitions and measurement methods of the candidate predictors and the outcome ascertainment criteria were standardized, and all research personnel received uniform training to minimize information bias.

All candidate predictors were obtained at the initial visit or during the first infusion, and outcomes were consistently observed through completion of the scheduled infusion on day 3. This ensured that information generated after outcome occurrence was not used as a model predictor.

During model development, all 11 prespecified candidate predictors were entered simultaneously into a multivariable logistic regression model, without data-driven variable selection based on *P* values from univariable analyses. Bootstrap resampling was used for internal validation to assess model overfitting and optimism. An independent cohort concurrently enrolled at the other study center was used for external validation to evaluate the model's generalizability across different clinical settings.

### 2.8 Statistical analysis and model development

Statistical analyses were performed using IBM SPSS Statistics version 28.0 and R software.

The supplementary functional-form analyses conducted during revision were performed in Python version 3.12.14 using NumPy version 2.3.5.

Continuous variables were summarized as the mean ± standard deviation or median (interquartile range), depending on their distributions, whereas categorical variables were summarized as frequencies and percentages. Baseline characteristics were described separately for the development and external validation cohorts. Standardized mean differences (SMDs) were used to assess differences in the distribution of patient characteristics between the two cohorts.

Early PIVC failure was treated as the binary dependent variable. All 11 prespecified candidate predictors were entered simultaneously into a multivariable logistic regression model, without selecting variables for model inclusion based on *P* values from univariable analyses. Before model development, variance inflation factors (VIFs) were calculated to assess multicollinearity among the candidate predictors.

All continuous predictors were entered into the multivariable logistic regression model using their original measurement units. Accordingly, age was modeled per 1-year increase, white blood cell count per 1 × 10⁹/L increase, serum albumin per 1-g/L increase, C-reactive protein per 1-mg/L increase, and hemoglobin per 1-g/L increase. For improved clinical interpretability and graphical presentation, selected effect estimates were additionally expressed on clinically meaningful scales: per 10-year increase for age, per 5-g/L increase for serum albumin, per 10-mg/L increase for C-reactive protein, and per 10-g/L increase for hemoglobin. These rescaled odds ratios were derived from the original regression coefficients without refitting the model. The difficult intravenous access score and number of special intravenous medication categories were expressed per 1-point and per 1-category increase, respectively. Categorical variables were included according to the prespecified coding schemes.

For each predictor, the regression coefficient (B), standard error (SE), Wald *χ*^2^ statistic, *P* value, odds ratio (OR), and 95% confidence interval (CI) were reported. All 11 prespecified predictors were retained in the final prediction model, and an individualized risk prediction equation was established using the regression coefficients and intercept.

The original model specified linear effects of continuous predictors on the log odds of early PIVC failure. A formal assessment of this assumption was subsequently undertaken as an additional analysis during revision. Age, white blood cell count, serum albumin, C-reactive protein, and hemoglobin were each modeled in turn using restricted cubic splines with three knots at their 10th, 50th, and 90th percentiles in the development cohort. Each spline model retained the other 10 predictors in their original forms and added one nonlinear parameter. A likelihood-ratio test with one degree of freedom compared each spline model with the original linear model. Serum albumin knots were 34.09, 41.30, and 48.11 g/L. For the discrete difficult intravenous access score and medication category count, indicator-variable models were separately compared with their linear-score specifications. These analyses were exploratory, with nominal *P* values reported without adjustment for multiple testing, and did not update the original prediction equation. A nonsignificant test was not interpreted as proof of linearity.

Internal validation was performed using 1,000 bootstrap resamples to quantify model optimism and obtain optimism-corrected performance estimates. Model discrimination was evaluated using the area under the receiver operating characteristic curve (AUC) with its 95% CI. Overall prediction error was assessed using the Brier score. Calibration performance was evaluated using the calibration intercept, calibration slope, and Emax. An intercept close to 0 and a slope close to 1 indicated good agreement between predicted and observed risks. Emax was used to quantify the maximum absolute deviation of the calibration curve from the ideal 45-degree line, with smaller values indicating better calibration. Calibration was also assessed graphically. In the development cohort, apparent and bootstrap bias-corrected calibration curves based on 1,000 bootstrap resamples were plotted against the ideal calibration line. In the external validation cohort, logistic and nonparametric calibration curves were plotted against the ideal calibration line. Rug marks along the *x*-axis were used to show the distribution of predicted probabilities.

For external validation, the regression coefficients and intercept derived from the development cohort were fixed and directly applied to the external validation cohort, without reselecting predictors or re-estimating model parameters. The AUC, Brier score, calibration intercept, and calibration slope were then evaluated in the external validation cohort.

Decision curve analysis (DCA) was performed to assess the model's clinical net benefit across a range of risk thresholds. The 95% confidence intervals for the estimated net benefit were calculated using 1,000 bootstrap resamples. In this context, the “treat-all” strategy assumed that all patients would be classified as high risk and undergo additional risk-oriented assessment or management, whereas the “treat-none” strategy assumed that no patients would receive such additional management beyond routine care. The net benefit of model-guided risk stratification was compared with these two reference strategies to evaluate its potential clinical utility. All statistical tests were two-sided, and *P* < 0.05 was considered statistically significant.

### 2.9 Ethical considerations

The study was approved by the Medical Ethics Committee of Shanghai General Hospital (Approval No.[2025]177) and was subsequently recognized and authorized by the external validation center, Guangzhong Road Community Health Service Center, Hongkou District, Shanghai. The study was conducted in accordance with the ethical principles of the Declaration of Helsinki. Before enrollment, all participants were fully informed of the study objectives and procedures, potential risks and benefits, confidentiality measures, and their right to participate voluntarily or withdraw from the study. Written informed consent was obtained from all participants or their legally authorized representatives, as appropriate.

## 3 Results

### 3.1 Comparison of baseline characteristics between the two cohorts

This dual-center prediction model study included 485 patients: 340 in the development cohort and 145 in the external validation cohort. The participant screening, eligibility assessment, exclusions, and inclusion in the development and external validation cohorts are shown in Figure 1.

**Figure 1:**
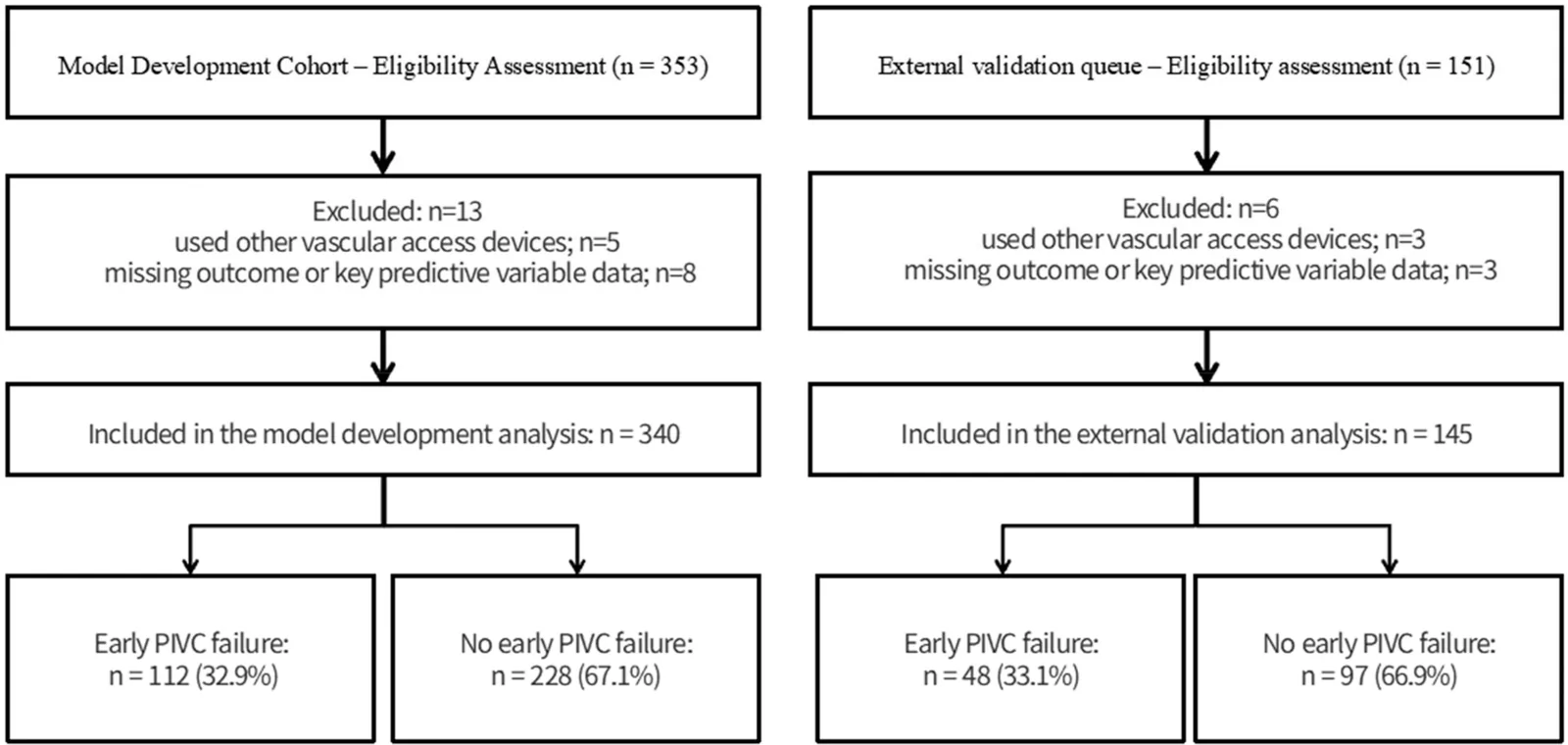
Participant flow diagram for the development and external validation cohorts.

Early PIVC failure occurred in 112 patients (32.9%) in the development cohort and 48 patients (33.1%) in the external validation cohort, indicating similar outcome event rates between the two cohorts. Eleven candidate predictors were included, with no missing data among any participants. The SMDs for all variables ranged from 0.003 to 0.116. The SMD was 0.116 for serum albumin and <0.10 for all other variables, indicating that the distributions of baseline characteristics were generally similar between the two cohorts, with only serum albumin showing an SMD slightly above 0.10. Baseline characteristics are presented in Table 1.

**Table 1:** Baseline characteristics of the development cohort and external validation cohort.

Variable	Development cohort (*n* = 340)	External validation cohort (*n* = 145)	SMD
Age, years	61.4 ± 15.6	61.2 ± 15.7	0.009
Male sex, *n* (%)	229 (67.4)	93 (64.1)	0.068
difficult intravenous access score	2.0 (1.0, 3.0)	2.0 (1.0, 3.0)	0.060
Number of categories of potentially irritating intravenous medications	3.0 (1.0, 3.0)	3.0 (1.0, 3.0)	0.078
White blood cell count, ×10⁹/L	9.43 (7.12, 12.46)	9.35 (6.71, 12.65)	0.084
Serum albumin, g/L	41.1 ± 5.1	41.7 ± 4.9	0.116
C-reactive protein, mg/L	15.1 (2.7, 56.8)	16.6 (4.0, 52.0)	0.031
Hemoglobin, g/L	123.5 ± 26.0	125.2 ± 23.8	0.066
PIVC placement in the dominant limb, *n* (%)	59 (17.4)	26 (17.9)	0.015
PIVC insertion at the dorsum of the hand, *n* (%)	329 (96.8)	138 (95.2)	0.081
History of diabetes mellitus, *n* (%)	38 (11.2)	19 (13.1)	0.059

Continuous variables are presented as mean ± standard deviation (SD) or median [interquartile range (IQR)], as appropriate; categorical variables are presented as *n* (%). SMD, standardized mean difference.

Each adverse event was assigned to only one primary failure cause. There were 112 adverse events in the training cohort, including 42 cases of phlebitis, 32 cases of infiltration/extravasation, 23 cases of catheter occlusion, 14 cases of catheter dislodgement/migration, and 1 case of local infection. A total of 48 adverse events were identified in the external validation cohort: 17 cases of phlebitis, 15 cases of infiltration/extravasation, 10 cases of catheter occlusion, 6 cases of catheter dislodgement/migration, and 0 case of local infection.

### 3.2 Multivariable logistic regression model development

All 11 prespecified candidate predictors were entered simultaneously into the multivariable logistic regression model. The VIFs ranged from 1.011 to 1.435, indicating no substantial multicollinearity. The model had a Nagelkerke R^2^ of 0.474.

Multivariable logistic regression analysis showed that the number of special intravenous medication categories, serum albumin, and catheter placement in the dominant limb had statistically significant regression coefficients (all *P* < 0.001). For each additional special intravenous medication category, the odds of early PIVC failure increased (OR=2.533, 95% CI: 1.755–3.657). For each 1-g/L increase in serum albumin, the odds of early PIVC failure decreased (OR=0.791, 95% CI: 0.739–0.847). The odds of early PIVC failure were higher among patients with catheter placement in the dominant limb than among those with catheter placement in the nondominant limb (OR=4.718, 95% CI: 2.244–9.920). The regression coefficients for age, sex, difficult intravenous access score, white blood cell count, C-reactive protein, hemoglobin, catheter insertion site, and history of diabetes mellitus were not statistically significant (all *P* > 0.05). The detailed regression parameters for each predictor are presented in Table 2, and the effect estimates with their 95% CIs are shown in Figure 2.

**Table 2:** Multivariable logistic regression analysis for early PIVC failure.

Predictor	B	SE	Wald *χ*^2^	*P value*	OR	95% CI	VIF
Age, per 1-year increase	−0.009	0.011	0.646	0.422	0.991	0.970–1.013	1.419
Male sex (male=1, female=0)	−0.167	0.314	0.284	0.594	0.846	0.458–1.564	1.107
Difficult intravenous access score, per 1-point increase	0.260	0.136	3.638	0.056	1.296	0.993–1.693	1.435
Number of special intravenous medication categories, per 1-category increase	0.930	0.187	24.642	<0.001	2.533	1.755–3.657	1.183
White blood cell count, per 1 × 10⁹/L increase	0.025	0.034	0.518	0.472	1.025	0.959–1.096	1.048
Serum albumin, per 1-g/L increase	−0.234	0.035	45.531	<0.001	0.791	0.739–0.847	1.053
C-reactive protein, per 1-mg/L increase	0.003	0.003	1.119	0.290	1.003	0.998–1.008	1.093
Hemoglobin, per 1-g/L increase	0.003	0.006	0.208	0.649	1.003	0.991–1.014	1.175
PIVC placement in the dominant limb (yes=1, no = 0)	1.551	0.379	16.738	<0.001	4.718	2.244–9.920	1.081
PIVC insertion outside the dorsum of the hand (outside=1, dorsum=0)	−1.360	0.980	1.926	0.165	0.257	0.038–1.751	1.011
History of diabetes mellitus (yes=1, no = 0)	−0.261	0.486	0.288	0.592	0.771	0.297–1.998	1.056
Intercept	5.567	1.685	10.919	0.001	261.559	—	—

Continuous predictors were entered in the units specified in the table. Male sex, PIVC placement in the dominant limb, and history of diabetes mellitus were coded as 1 for yes and 0 for no. For insertion site, the dorsum of the hand was coded as 0 and other sites as 1; the reported OR therefore compares other sites with the dorsum of the hand. All 11 prespecified candidate predictors were retained. B, regression coefficient; SE, standard error; OR, odds ratio; CI, confidence interval; VIF, variance inflation factor. *P* values displayed as 0.000 are reported as <0.001.

**Figure 2:**
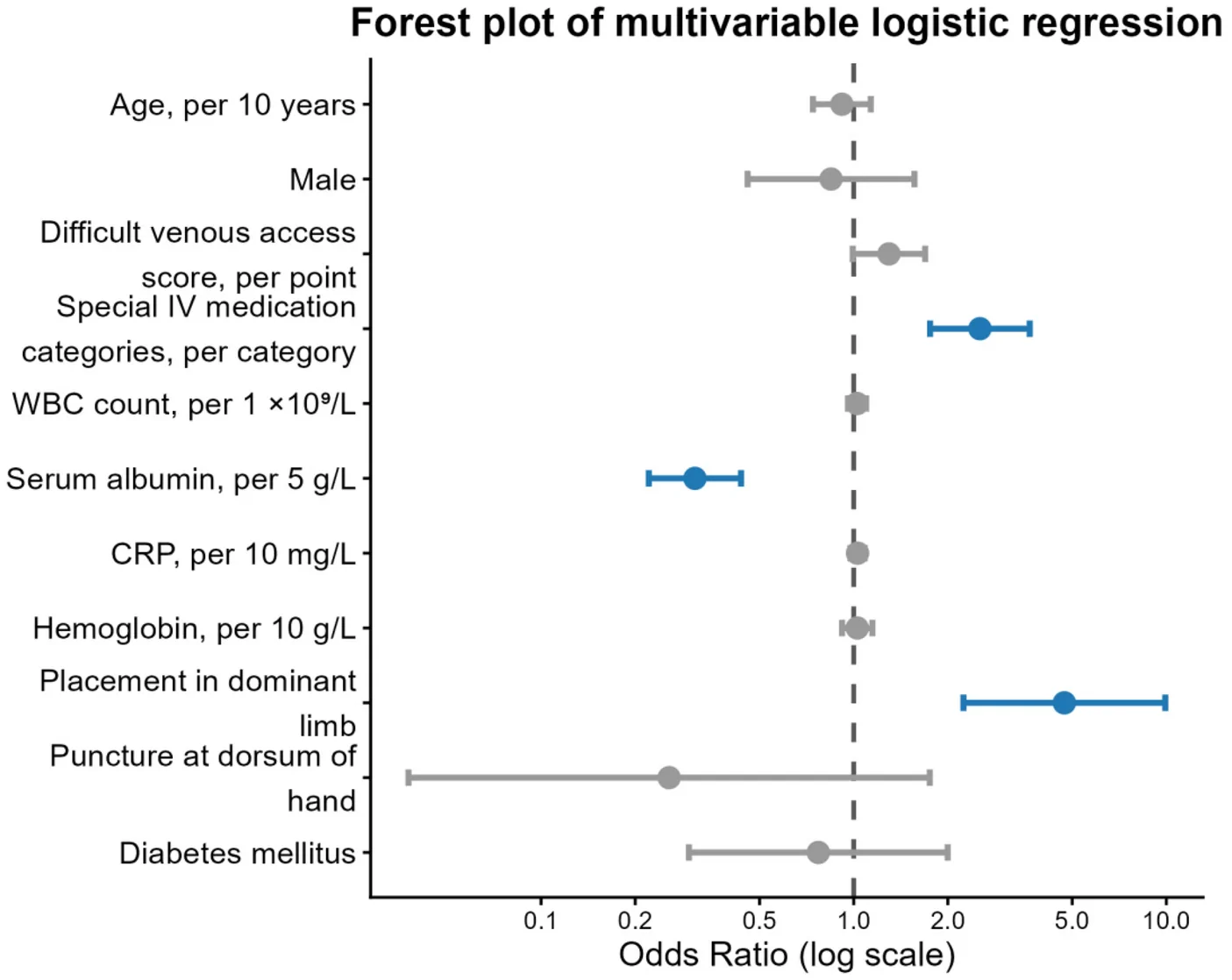
Forest plot of the multivariable logistic regression analysis.

In the supplementary functional-form analyses, no statistically significant departure from linearity was detected for serum albumin (likelihood-ratio chi-square=0.833, 1 df; *P* = 0.361) or hemoglobin (*P* = 0.685). Nonlinear associations were detected for age (*P* = 0.019), white blood cell count (*P* = 0.014), and C-reactive protein (*P* = 0.001). The indicator-variable comparisons did not detect a departure from the linear-score specification for difficult intravenous access (*P* = 0.167), but did detect a departure for medication category count (*P* = 0.022). Thus, these exploratory analyses did not provide strong evidence against the linear specification for serum albumin, but indicated possible functional-form misspecification for other predictors. The original model was retained for the reported prediction equation and validation analyses; a model incorporating alternative functional forms would require renewed internal and external validation.

All 11 prespecified predictors were retained in the final model. Using the original measurement units and the binary coding specified in Table 2, the model prediction equation, with coefficients rounded to three decimal places, was as follows: Logit(P) = 5.567−0.009 × age−0.167 × male sex + 0.260 × difficult intravenous access score + 0.930 × number of special intravenous medication categories + 0.025 × white blood cell count−0.234 × serum albumin + 0.003 × C-reactive protein + 0.003 × hemoglobin + 1.551 × catheter placement in the dominant limb−1.360 × catheter insertion outside the dorsum of the hand−0.261 × history of diabetes mellitus.

### 3.3 Model predictive performance and validation

Model discrimination was evaluated using the area under the receiver operating characteristic curve (AUC). The apparent AUC was 0.862 (95% CI: 0.823–0.901) in the development cohort and was 0.840 after correction using 1,000 bootstrap resamples. In the external validation cohort, the AUC was 0.845 (95% CI: 0.779–0.910). The relatively small difference between the apparent and bootstrap-corrected AUCs suggested limited optimism in the development cohort. Moreover, discrimination was maintained in the external validation cohort, indicating that the model maintained good discrimination in an independent external population (Table 3 and Figure 3).

**Table 3:** Discrimination, brier score, and calibration measures of the prediction model.

Evaluation stage	AUC (95% CI)	Brier score	Calibration intercept	Calibration slope
Development cohort (apparent)	0.862 (0.823–0.901)	0.140	0.000	1.000
Development cohort (bootstrap-corrected)	0.840	0.154	−0.065	0.849
External validation cohort	0.845 (0.779–0.910)	0.151	0.081	0.850

AUC, area under the receiver operating characteristic curve; CI, confidence interval. Internal validation was performed using 1,000 bootstrap resamples. The 95% confidence intervals for the apparent development-cohort and external-validation AUCs were estimated using bootstrap resampling.

**Figure 3:**
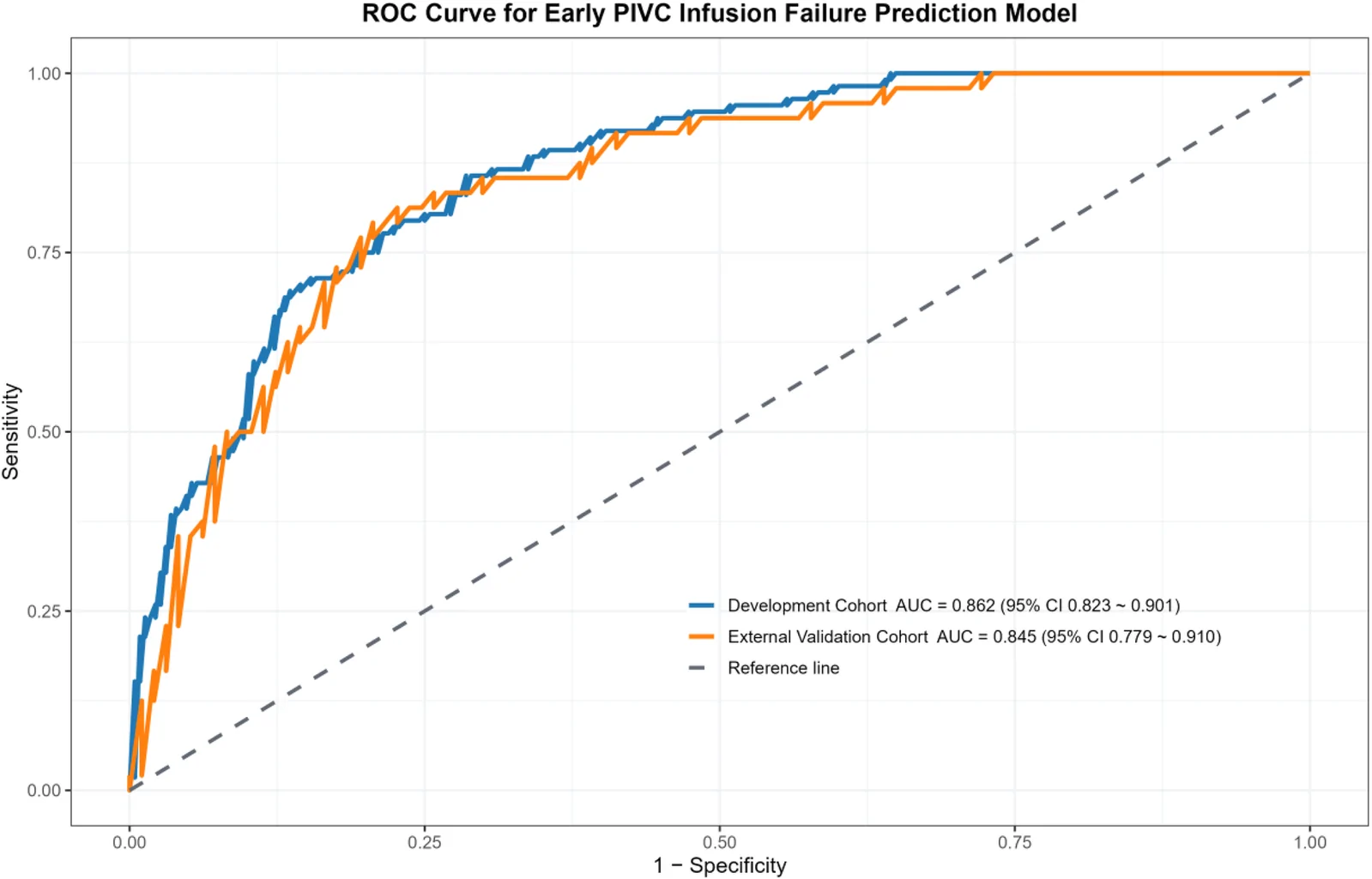
Receiver operating characteristic (ROC) curves of the risk prediction model for early PIVC failure. The blue curve represents the development cohort (AUC=0.862, 95% CI: 0.823–0.901); the orange curve represents the external validation cohort (AUC=0.845, 95% CI: 0.779–0.910); and the gray dashed line represents the reference line indicating no discriminative ability.

Overall prediction error and calibration performance were further evaluated using the Brier score, calibration intercept, calibration slope, and Emax. In the development cohort, the apparent Brier score was 0.140, with a calibration intercept of 0.000 and a calibration slope of 1.000. After correction using 1,000 bootstrap resamples, the Brier score was 0.154, the calibration intercept was −0.065, the calibration slope was 0.849, and Emax was 0.067. In the external validation cohort, the Brier score was 0.151, the calibration intercept was 0.081, the calibration slope was 0.850, and Emax was 0.096. The bootstrap-corrected development-cohort and external-validation calibration intercepts were close to zero, suggesting limited systematic over- or underprediction. However, the bootstrap-corrected calibration slope of 0.849 and the external-validation slope of 0.850 were below the ideal value of 1, indicating some overfitting and that predicted risks tended to be too extreme. The Emax values of 0.067 and 0.096 further indicated non-negligible deviations from ideal calibration. Thus, although calibration remained reasonably close to the ideal reference, some miscalibration was evident in both cohorts and should be considered when interpreting individual predicted probabilities (Table 3 and Figure 4).

**Figure 4:**
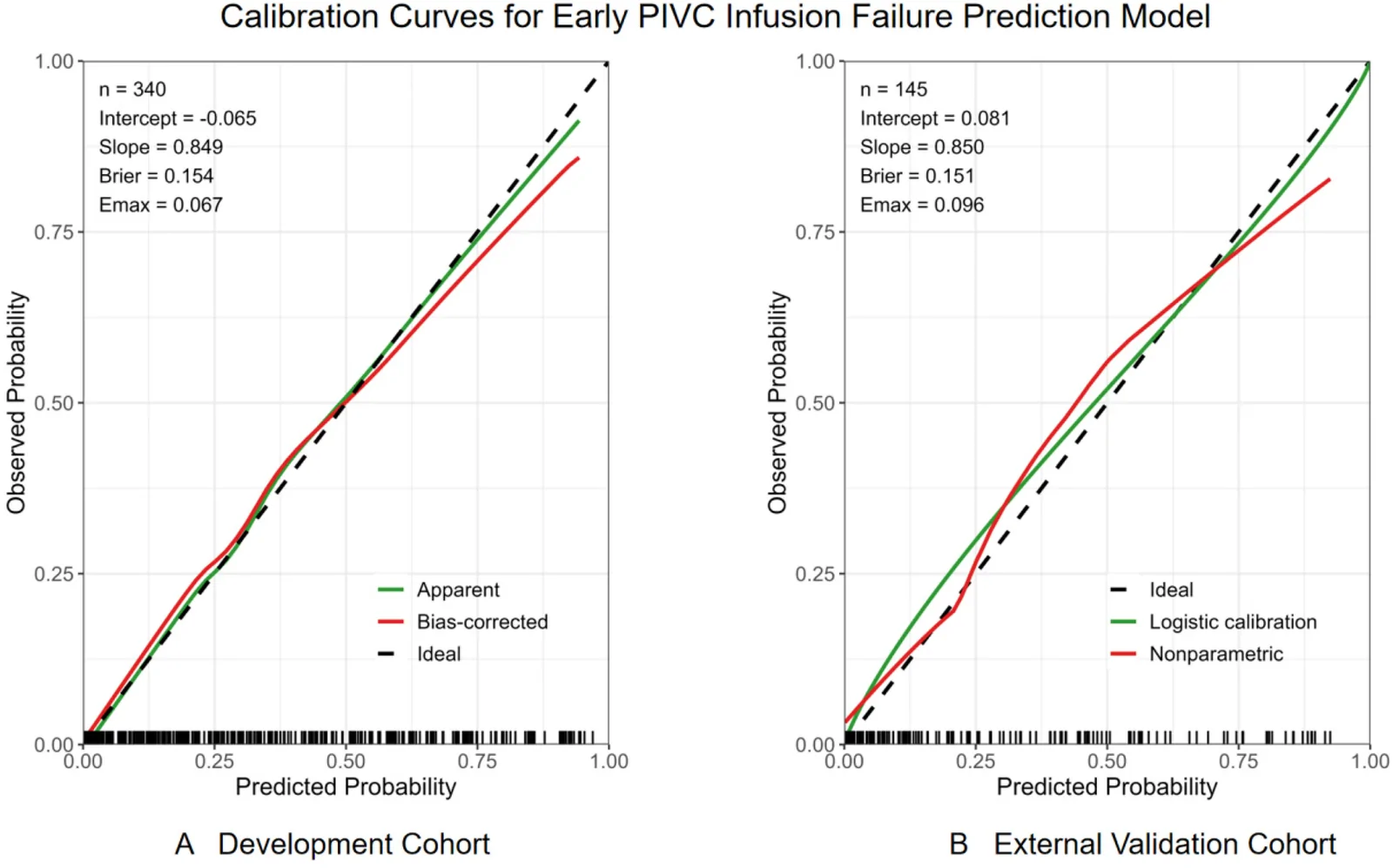
Calibration curves of the early PIVC infusion failure prediction model in the development and external validation cohorts. **(A)** Development cohort (*n* = 340), showing the apparent calibration curve (green), bootstrap bias-corrected calibration curve based on 1,000 resamples (red), and ideal calibration line (black dashed). The reported calibration intercept, slope, Brier score, and Emax correspond to the bootstrap-corrected estimates. **(B)** External validation cohort (*n* = 145), showing the logistic calibration curve (green), nonparametric calibration curve (red), and ideal calibration line (black dashed). The reported calibration intercept, slope, Brier score, and Emax correspond to the external validation estimates. Rug marks along the *x*-axis show the distribution of predicted probabilities.

### 3.4 Decision curve analysis

DCA was used to evaluate the model's potential clinical utility, as shown in Figure 5. Across a broad range of threshold probabilities, the model generally yielded a higher net benefit in both the development and external validation cohorts than the treat-all and treat-none strategies. This suggests that model-guided identification of patients who may benefit from additional risk-oriented assessment or management could provide greater clinical net benefit than either applying the additional strategy to all patients or to none. The net benefit gradually decreased as the threshold probability increased and approached, and eventually fell below, zero in the external validation cohort at the highest threshold probabilities. The bootstrap 95% confidence intervals widened at higher threshold probabilities, particularly in the external validation cohort, indicating greater uncertainty in the estimated net benefit in this range. Overall, the model demonstrated potential clinical utility across a clinically relevant range of threshold probabilities, although the benefit became less certain at higher thresholds.

**Figure 5:**
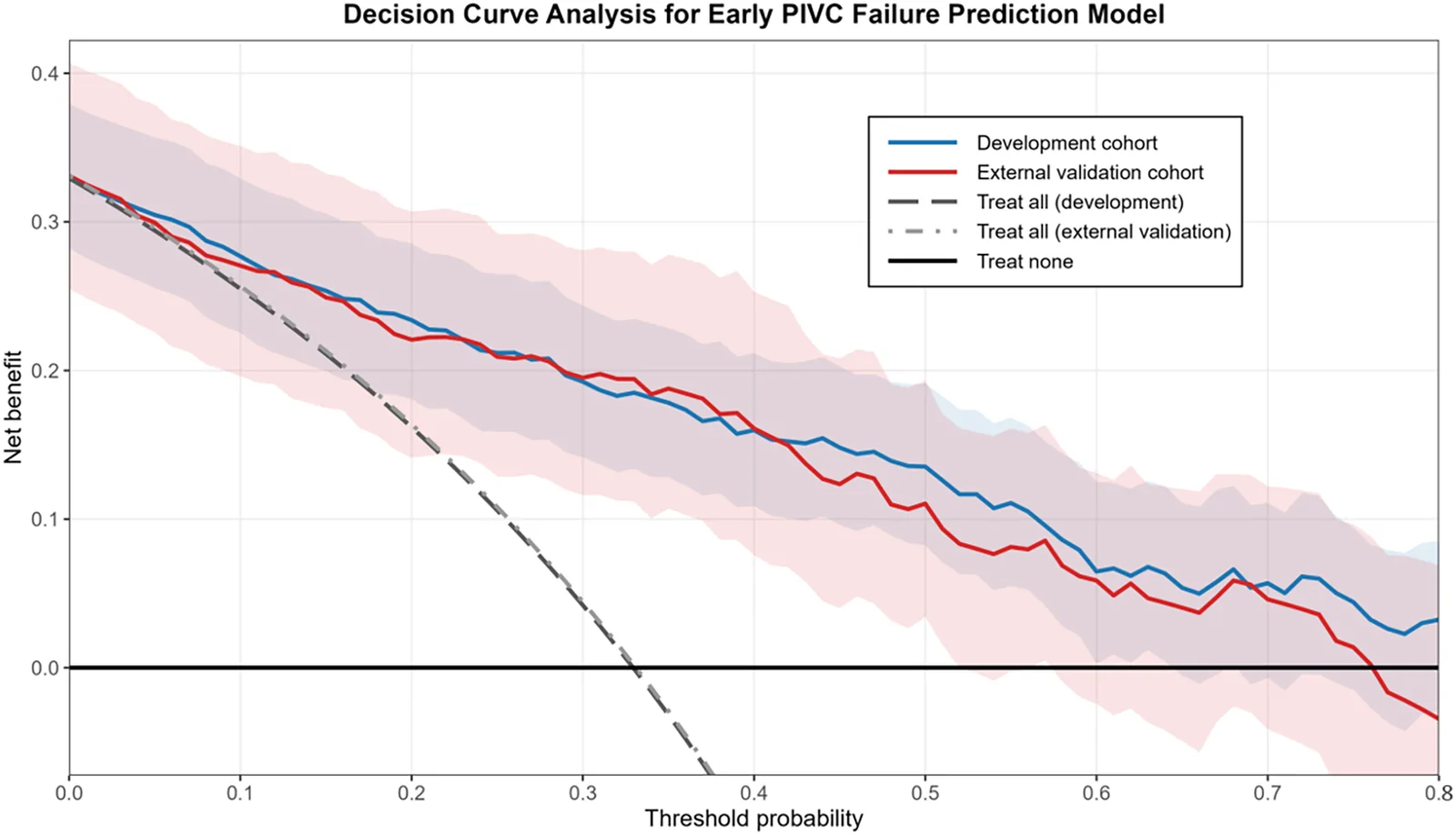
Decision curve analysis for early PIVC infusion failure prediction model. The blue and red solid lines represent the net benefit of the prediction model in the development and external validation cohorts, respectively. Shaded areas indicate 95% confidence intervals estimated using 1,000 bootstrap resamples. The dark-gray long-dashed and light-gray dot-dashed lines represent the treat-all strategies in the development and external validation cohorts, respectively, while the black solid line represents the treat-none strategy. A higher net benefit than both reference strategies indicates potential clinical utility within the corresponding threshold-probability range.

## 4 Discussion

### 4.1 Early PIVC failure and its predictive value Among outpatients and emergency department patients receiving infusion therapy on consecutive days

The incidence of early PIVC failure was 32.9% in the development cohort and 33.1% in the external validation cohort, with similar rates observed across the two study centers. Kassahun et al. (9) prospectively evaluated the first PIVC inserted in adult medical and surgical inpatients and found that PIVC failure occurred in 124 of 418 patients, corresponding to an incidence of 29.7%, which was similar to that observed in the present study. Blanco-Mavillard et al. (10) evaluated 711 PIVCs in hospitalized adult patients in Spain and found that 297 PIVCs failed and required unplanned removal, corresponding to a failure rate of 41.8%. Kache et al. (11) further reported in a study involving 90,743 PIVCs that the failure rates were 53.1% for PIVCs inserted in the emergency department and 53.4% for those inserted in inpatient units. The mean dwell time of PIVCs inserted in the emergency department was approximately 3.4 days. More recently, Bakcek Akcelik and Ayhan specifically examined PIVC-related phlebitis and infiltration among patients in an emergency department, further highlighting the clinical relevance of catheter-related complications in this population (7). Their findings support the importance of early risk identification and targeted catheter assessment and management in emergency department practice. Differences in failure rates across studies may be attributable to variations in patient disease profiles, clinical settings, infused medications, catheter dwell times, and criteria used to define failure. Notably, previous studies have generally evaluated PIVC failure throughout the entire catheter dwell period, whereas the present study focused only on the short-term treatment period from initial catheter insertion to completion of the scheduled infusion on day 3. Nevertheless, approximately one-third of patients experienced PIVC failure, indicating that loss of catheter function is not limited to prolonged dwell and that early maintenance of catheter function in outpatient and emergency department settings also warrants attention.

Early PIVC failure may result from the combined effects of chemical irritation, mechanical injury, and local inflammatory responses. Venipuncture and catheter insertion can damage the vascular endothelium, while the continued presence of the catheter within the vessel and small catheter movements caused by limb activity may produce further mechanical irritation. Irritant infusates with high osmolarity or strong acidity may exacerbate endothelial injury and local inflammation. Persistent vascular wall injury, inflammation, and coagulation responses may subsequently lead to phlebitis, catheter occlusion, infiltration, or extravasation, ultimately resulting in loss of catheter function (12). Previous studies have found that the infusion of irritant solutions, catheter insertion site, and factors related to catheter use are associated with PIVC failure, further demonstrating its multifactorial nature (13, 14).

This issue has particular clinical significance for outpatients and emergency department patients receiving infusion therapy on consecutive days. Hospitalized patients can receive continuous observation from healthcare professionals while a PIVC remains in place. In contrast, after completing the first infusion, outpatients and emergency department patients spend most of the catheter dwell period outside the healthcare facility, making catheter protection and the recognition of abnormalities more dependent on patients and their family members. If the catheter fails before subsequent treatment, the patient still requires repeat venipuncture despite having already incurred the cost of PIVC placement and experienced inconvenience during physical activity, washing, and other daily activities while carrying the catheter. Previous randomized studies have shown that unnecessary repeated PIVC replacement increases the number of catheter insertions, use of medical supplies, and patients’ burden of invasive procedures (15). Therefore, for outpatients and emergency department patients receiving infusion therapy for three consecutive days, PIVC management should focus not only on prolonging catheter dwell time but also on whether the initially inserted catheter can remain functional until completion of the planned treatment course.

The present study further integrated multidimensional clinical information available during the first infusion to generate individualized predicted probabilities. The apparent AUC was 0.862 in the development cohort, decreasing to 0.840 after bootstrap correction, while the AUC was 0.845 in the external validation cohort. However, the bootstrap-corrected calibration slope of 0.849 and external-validation slope of 0.850 indicated some overfitting, with predicted risks tending to be too extreme. Although the calibration intercepts were close to zero, these findings indicate that discrimination was more robust than calibration and that individual predicted probabilities should be interpreted with caution. The prediction model for PIVC-related phlebitis developed by Welvaars et al. (16) achieved AUCs of 0.89 in the test set and 0.72 during external validation, suggesting that integrating clinical factors to predict the risk of PIVC-related complications is feasible. Unlike previous studies, the present study focused on the specific clinical period from completion of the first infusion to completion of treatment on day 3 among outpatients and emergency department patients and externally validated the model at an independent healthcare institution. The significance of this study lies not merely in achieving a high AUC but in shifting risk identification to the period before patients are discharged with a PIVC in place, thereby providing nurses with a quantitative basis for moving beyond routine catheter assessment toward early risk stratification.

### 4.2 Clinical significance of special intravenous medications, serum albumin, and catheter placement in the dominant limb

The present study showed that the number of special intravenous medication categories, serum albumin, and catheter placement in the dominant limb were important predictors of early PIVC failure. These factors reflect treatment-related chemical irritation, the patient's underlying physiological condition, and mechanical exposure after catheter placement, respectively, suggesting that early failure may result from the combined effects of medications, host factors, and the catheter-use environment.

The strong association between the number of special intravenous medication categories and early PIVC failure is biologically plausible. In the present study, special intravenous medications included hyperosmolar medications, pH-related irritant medications, and particulate/excipient-related irritant medications. Manrique-Rodríguez et al. (17) evaluated 307 preparation regimens involving 112 commonly used intravenous medications and found that many intravenous preparations exhibited physicochemical characteristics, such as high osmolarity or extreme pH, that may increase venous irritation. Similarly, Ballesteros-Peña et al. (18) reported that physicochemical properties including pH and osmolarity may contribute to local venous irritation. A multicenter study by Ferraz-Torres et al. (19) further demonstrated an association between intravenous medication burden and chemical phlebitis. Potential mechanisms may include osmotic injury to vascular endothelial cells under hyperosmolar conditions, disruption of endothelial homeostasis by pH values outside the physiological range, and local inflammatory responses related to formulation-, preparation-, or infusion system-associated particulates as well as certain excipients. However, the risk of tissue injury cannot be fully determined on the basis of a single physicochemical parameter alone (20). Therefore, the number of special intravenous medication categories in this study is more appropriately interpreted as an integrated indicator of treatment-related venous irritation burden. In clinical nursing practice, when patients are exposed to multiple categories of potentially irritating intravenous medications, nurses should comprehensively evaluate the infusion regimen, venous condition, and appropriateness of the vascular access device, rather than focusing solely on whether successful catheter insertion can be achieved.

Serum albumin, in contrast, may primarily reflect the patient's underlying physiological condition. Soeters et al. (21) noted that hypoalbuminemia is associated not only with nutritional status but also with systemic inflammatory responses and altered capillary permeability. Therefore, lower serum albumin levels may indicate a greater inflammatory burden, an increased tendency toward tissue edema, and reduced physiological reserve. A large-sample meta-analysis by Valeriani et al. (22) also found that hypoalbuminemia was associated with an increased risk of venous thromboembolism (VTE), suggesting that albumin levels may be related to coagulation and the vascular endothelial environment. Although these mechanisms do not directly demonstrate that hypoalbuminemia causes PIVC failure, they provide a plausible explanation for the association observed in the present study. Importantly, our findings do not imply that albumin supplementation can reduce the risk of PIVC failure. Rather, serum albumin should be interpreted as a readily available indicator of the patient's underlying condition at the initial visit and used in conjunction with medication characteristics, venous conditions, and other relevant factors for risk assessment.

The association between catheter placement in the dominant limb and early PIVC failure may be more closely related to mechanical exposure. However, findings from previous studies have been inconsistent. Furlan et al. (23) summarized evidence linking catheter placement in the dominant limb to phlebitis,whereas Privitera et al. (24) found no significant association between the dominant limb and catheter-related complications in patients with acute stroke. After returning home, outpatients and emergency department patients frequently use their dominant limb for activities such as eating, dressing, using a mobile phone, carrying objects, and washing. This greater activity may increase minor catheter movement, tension on the dressing, and mechanical friction between the catheter and the vessel wall, thereby increasing the likelihood of catheter migration, infiltration, or occlusion. Nevertheless, given the large magnitude of effect observed in our study (OR=4.718), an alternative interpretation needs to be considered: clinicians may preferentially select the dominant limb for cannulation when the veins of the non-dominant limb are of poor quality or inaccessible. Under this scenario, poorer local venous status or higher cannulation difficulty, rather than limb dominance itself, could contribute to elevated early PIVC failure risk. Although we adjusted for the global difficult intravenous access score in the multivariable model, this score reflects overall venous access conditions and cannot capture side-specific differences in vein quality between dominant and non-dominant limbs. Side-by-side venous assessment data for bilateral extremities were not collected in this study; hence residual confounding originating from inter-limb venous discrepancies cannot be completely ruled out.

However, because out-of-hospital limb activity and catheter microdisplacement were not directly measured in this study, these proposed mechanisms require further investigation. Catheter placement in the dominant limb should therefore not be considered an absolute contraindication in clinical practice. Instead, when treatment requirements permit, both limb dominance and side-specific local vein quality should be considered when selecting the insertion site and providing catheter-care education to patients discharged with a PIVC in place.

### 4.3 From risk identification to stratified management: implications of the model for discharge With a PIVC in place

The clinical value of the model does not lie in using a predicted probability to directly determine whether a patient's PIVC should be retained or removed. Rather, it provides a basis for risk stratification when deciding whether a patient should be discharged with the PIVC in place after the first infusion. PIVC failure is multifactorial, and the predominant source of risk may differ among patients, arising from medication-related irritation, underlying physiological condition, or mechanical factors. Therefore, even patients with similar predicted probabilities may require different nursing priorities. The international e-Delphi consensus on the prevention of PIVC failure developed by Blanco-Mavillard et al. (25) similarly emphasized that preventing catheter failure requires attention to multiple aspects, including vascular access appropriateness, catheter insertion, securement and maintenance, catheter removal, and organizational management, and that no single intervention is suitable for all patients. Therefore, the model is better suited as a tool for identifying patients who require further assessment rather than for directly prescribing a specific intervention.

For patients with a low predicted risk and a normally functioning catheter, an important benefit of retaining the PIVC for subsequent short-term treatment is the avoidance of unnecessary repeat venipuncture. In a cluster-randomized trial involving 1,198 adults, Xu et al. (26) found no significant differences in the incidence of phlebitis, occlusion, infiltration, or accidental removal between clinically indicated PIVC replacement and routine replacement every 72–96 h. The study by Kang et al. (27) also suggested that clinically indicated replacement can reduce the number of catheter insertions without compromising patient safety. Although these studies primarily involved hospitalized patients, they consistently support the principle that unnecessary invasive procedures should not be performed solely on the basis of a standardized time interval when intravenous therapy remains necessary and the catheter is functioning normally. For low-risk outpatients and emergency department patients in the present study, retaining the PIVC to complete the short-term treatment course may better fulfill the original objective of reducing repeated venipuncture.

For high-risk patients, individualized management should be implemented according to the predominant source of risk. When risk is primarily related to irritant medications, the medication characteristics, infusion concentration and rate, venous conditions, and compatibility of the existing PIVC with the prescribed treatment regimen should be reassessed. When mechanical factors are more prominent, attention should focus on optimizing vein and insertion-site selection, catheter securement, and protection during physical activity. Importantly, selecting a suitable vein with relatively adequate blood flow does not imply using a larger-bore catheter. The smallest catheter size capable of meeting the treatment requirements should remain the preferred option. The Dressings and Securements for the Prevention of Peripheral Intravenous Catheter Failure in Adults (SAVE) randomized trial conducted by Rickard et al. (28) and the systematic review by Ray-Barruel et al. (29) both suggested that catheter stabilization, standardized maintenance, and care bundles may influence PIVC outcomes. However, no single standardized intervention is suitable for all patients. Therefore, nursing interventions following risk stratification should be tailored to each patient's specific sources of risk rather than applying the same management approach to all high-risk patients.

Because outpatients and emergency department patients spend most of the catheter dwell period outside the healthcare facility, patient education is also an essential component of stratified management. Laan et al. (30) found that some patients with PIVCs did not fully understand the purpose of catheter retention or how to respond to catheter-related abnormalities. Doll et al. (31) also identified variations in nursing professionals’ knowledge of catheter stabilization, flushing, insertion-site assessment, and complication recognition. For patients discharged with a PIVC in place, particularly those predicted to be at high risk, health education should include specific guidance on protecting the dressing and catheter, avoiding traction and repeated limb flexion, recognizing pain, redness, swelling, leakage, or infusion abnormalities, and seeking timely medical attention. These findings support a care pathway of “risk prediction–nursing review–stratified management–dynamic reassessment.” In the absence of other contraindications, low-risk patients may receive routine management with the PIVC retained, whereas high-risk patients may require enhanced vascular access assessment, catheter securement and protection, or health education tailored to their specific sources of risk. Catheter status should still be reassessed before each subsequent infusion to ensure that a single risk prediction does not replace ongoing clinical judgment.

In practical terms, identification of a patient as high risk would not automatically mandate catheter removal or replacement. Rather, it could trigger a focused nursing review, including reassessment of catheter function and vascular access appropriateness, reinforcement of catheter securement and protection, individualized education regarding catheter care and warning signs, and, when appropriate, reconsideration of whether continued PIVC retention or an alternative vascular access strategy is most suitable for the planned infusion therapy.

DCA indicated that the model may provide clinical net benefit across a range of threshold probabilities; however, this study did not directly determine whether model-guided nursing care reduces PIVC failure. At present, the model is therefore better suited as an auxiliary tool for risk stratification and resource allocation in outpatient and emergency nursing practice. Future studies could develop nursing pathways corresponding to different risk levels and conduct prospective impact studies to evaluate whether model-guided care reduces unplanned catheter removal and repeated venipuncture, alleviates additional patient burden, and improves patients’ experience of intravenous therapy.

## 5 Conclusions

Using data from a dual-center prospective cohort, this study developed and externally validated a risk prediction model for early PIVC failure among outpatients and emergency department patients receiving infusion therapy on consecutive days. The model incorporated 11 clinical indicators available during the first infusion and showed promising discrimination, while the calibration findings, limited validation sample, and exploratory evidence of nonlinear predictor effects indicate a need for further refinement and independent evaluation. The number of special intravenous medication categories, serum albumin, and catheter placement in the dominant limb were important predictors. This model may serve as a supportive tool for early risk stratification and individualized PIVC management before patients are discharged with a PIVC in place. It is intended to complement, rather than replace, ongoing catheter assessment and professional clinical judgment.

## 6 Limitations

This study has several limitations. First, although the model underwent independent external validation, the study was conducted at only two centers and was limited to outpatients and emergency department patients receiving intravenous infusion therapy for three consecutive days. Both cohorts were drawn from Hongkou District, Shanghai, and showed similar measured baseline characteristics (SMDs, 0.003–0.116). The shared catchment area, hospital triage process, and identical eligibility criteria may partly explain this similarity. Therefore, the external validation primarily assessed model performance across institutions within the same district and provided limited evidence of transportability to geographically or clinically different populations. Further validation in different geographic regions, healthcare systems, and settings with varying infusion and vascular access practices is warranted before broader clinical implementation. Validation in patients undergoing longer treatment courses would also help determine the model's applicability beyond the current study population. Second, the available sample size warrants consideration. The development cohort contained 112 events for 11 predictor parameters. The bootstrap-corrected calibration slope of 0.849 and the external-validation slope of 0.850 suggest some overfitting and overly extreme risk predictions. Bootstrap validation quantified optimism but did not increase the information available for model estimation. Furthermore, the external validation cohort contained only 48 events, limiting the precision of the performance estimates, particularly those for calibration and clinical net benefit. Larger independent cohorts, with sample sizes planned according to the desired precision of these measures, are needed. Further recalibration may also be necessary before the model is applied to populations that differ from the present cohorts. Third, the distribution and measurement of catheter-placement variables posed several limitations. Catheter insertion site was highly imbalanced, with most catheters inserted on the dorsum of the hand and only a small number inserted at other sites. Consequently, the coefficient for insertion outside the dorsum of the hand was imprecisely estimated, as reflected by its wide 95% confidence interval (OR=0.257, 95% CI: 0.038–1.751). Because this coefficient was retained in the final prediction equation, individual predicted risks may be sensitive to this unstable estimate and should therefore be interpreted with caution. The insertion-site coefficient should not be used in isolation to guide clinical decisions. Larger datasets with adequate representation of non-dorsum insertion sites, together with coefficient shrinkage or penalized modeling and further external validation, are needed to assess and stabilize this effect. In addition, only a global difficult intravenous access score was included, and venous conditions were not assessed separately for the dominant and non-dominant extremities. Therefore, we could not determine whether the higher failure risk associated with dominant-limb cannulation arose from mechanical stress related to greater limb activity or from residual confounding caused by differences in local vein quality between the two extremities. Future studies should record venous conditions separately for both extremities to distinguish these competing explanations. Fourth, catheter-related events occurring at home between visits were partly ascertained through patient reports obtained during the subsequent visit or by telephone interview, rather than through direct observation by trained healthcare professionals. Therefore, unreported catheter-related symptoms or problems may have been missed or misclassified. To reduce this risk, patient-reported information was integrated with standardized nursing assessment, and uncertain outcome classifications were independently reviewed by a vascular access specialist nurse. Fifth, special intravenous medications were summarized according to the number of medication categories, without further accounting for specific medications, concentrations, infusion rates, or cumulative exposure. Therefore, the medication-category count should be interpreted as a general indicator of treatment-related venous irritation burden rather than as an estimate of the effect of any specific medication or administration regimen. Several potentially relevant catheter- and procedure-related factors, such as catheter gauge, number of insertion attempts, securement practices, and flushing practices, were also not included as candidate predictors. These factors may influence PIVC failure and may vary across institutions and clinical settings. Their omission may therefore affect model performance and transportability when the model is applied in settings with different catheter insertion and maintenance practices. Future studies should evaluate these factors and further validate the model across settings with diverse vascular access practices. Sixth, continuous predictors were initially modeled using linear terms. The additional analyses conducted during revision did not detect significant nonlinearity for serum albumin or hemoglobin but identified nonlinear signals for age, white blood cell count, and C-reactive protein, as well as a departure from a linear effect of medication category count. These exploratory findings were subject to multiple testing and limited by the available sample size, and the absence of a statistically significant result does not establish linearity. Retaining the original functional forms may therefore have resulted in residual model misspecification. Future model refinement should consider flexible predictor effects, account for the resulting additional parameters in sample-size planning, and repeat both internal and external validation.

Finally, this study focused primarily on model development and external validation. A prospective impact study has not yet been conducted to determine whether model-guided nursing care can reduce PIVC failure, repeated venipuncture, and patient burden in clinical practice. Therefore, the model should currently be regarded as a supportive risk-stratification tool rather than a replacement for ongoing catheter assessment and professional clinical judgment. Its clinical effectiveness requires further investigation.

## Data Availability

The raw data supporting the conclusions of this article will be made available by the authors, without undue reservation.

## References

[1] Marsh N Larsen EN Ullman AJ Mihala G Cooke M Chopra V. Peripheral intravenous catheter infection and failure: a systematic review and meta-analysis. Int J Nurs Stud. (2024) 151:104673. 10.1016/j.ijnurstu.2023.104673 38142634

[2] Marsh N Webster J Ullman AJ Mihala G Cooke M Chopra V. Peripheral intravenous catheter non-infectious complications in adults: a systematic review and meta-analysis. J Adv Nurs. (2020) 76(12):3346–62. 10.1111/jan.14565 33016412

[3] Marsh N Webster J Larsen E Cooke M Mihala G Rickard CM. Observational study of peripheral intravenous catheter outcomes in adult hospitalized patients: a multivariable analysis of peripheral intravenous catheter failure. J Hosp Med. (2018) 13(2):83–9. 10.12788/jhm.2867 29073316

[4] Chen Y- Fan X- Liu M- Wang J Yang Y- Su Y-. Risk factors for peripheral venous catheter failure: a prospective cohort study of 5,345 patients. J Vasc Access. (2022) 23(6):911–21. 10.1177/11297298211015035 33985394 PMC9585540

[5] Nickel B Gorski L Kleidon T Kyes A DeVries M Keogh S. Infusion therapy standards of practice, 9th edition. J Infus Nurs. (2024) 47(1S Suppl 1):S1–s285. 10.1097/NAN.0000000000000532 38211609

[6] Yasuda H Rickard CM Mimoz O Marsh N Schults JA Drugeon B. Development of a machine learning-based model for predicting the incidence of peripheral intravenous catheter-associated phlebitis. J Crit Care Med (Targu Mures). (2024) 10(3):232–44. 10.2478/jccm-2024-0028 39108413 PMC11295139

[7] Bakcek Akcelik Ö Ayhan H. Peripheral intravenous catheter-related phlebitis and infiltration in an emergency department: a descriptive study. J Infus Nurs. (2024) 47(3):155–62. 10.1097/NAN.0000000000000548 38744240

[8] van Loon FHJ Van Hooff LWE De Boer HD Koopman SSHA Buise MP Korsten HHM. The modified A-DIVA scale as a predictive tool for prospective identification of adult patients at risk of a difficult intravenous access: a multicenter validation study. J Clin Med. (2019) 8(2):144. 10.3390/jcm8020144 30691137 PMC6406455

[9] Kassahun CW Abate AT Tezera ZB Beshah DT Agegnehu CD Getnet MA. Incidence and associated factors of failed first peripheral intravenous catheters among adult patients at medical surgical wards in public referral hospitals of west amhara, Ethiopia, 2021. Nurs Res Pract. (2022) 2022:8261225. 10.1155/2022/8261225 35103104 PMC8800617

[10] Blanco-Mavillard I Rodríguez-Calero MÁ De Pedro-Gómez J Parra-García G Fernández-Fernández I Castro-Sánchez E. Incidence of peripheral intravenous catheter failure among inpatients: variability between microbiological data and clinical signs and symptoms. Antimicrob Resist Infect Control. (2019) 8:124. 10.1186/s13756-019-0581-8 31367345 PMC6647274

[11] Kache S Patel S Chen N-W Qu L Bahl A. Doomed peripheral intravenous catheters: bad outcomes are similar for emergency department and inpatient placed catheters: a retrospective medical record review. J Vasc Access. (2022) 23(1):50–6. 10.1177/1129729820974259 33234001

[12] Zhu LL Wang YH Zhou Q. Progress in research on the mechanisms and interventions of phlebitis from the perspective of vascular endothelial cell and signaling pathway. J Inflamm Res. (2023) 16:6469–81. 10.2147/JIR.S450149 38170089 PMC10759916

[13] Bahl A Millard M Hijazi M Chen N-W. Predictors of ultrasound-guided peripheral intravenous catheter failure: a secondary analysis of existing trial data. J Vasc Access. (2024) 25(2):519–25. 10.1177/11297298221122118 36113061

[14] Jiménez-Martínez E Adamuz J González-Samartino M Muñoz-Carmona MA Hornero A Martos-Martínez MP. Peripheral intravenous catheter failure, nurse staffing levels and care complexity individual factors: a retrospective multicentre cohort study. PLoS One. (2024) 19(5):e0303152. 10.1371/journal.pone.0303152 PMC11081384 38722995

[15] Rickard CM Webster J Wallis MC Marsh N McGrail MR French V. Routine versus clinically indicated replacement of peripheral intravenous catheters: a randomised controlled equivalence trial. Lancet. (2012) 380(9847):1066–74. 10.1016/S0140-6736(12)61082-4 22998716

[16] Welvaars K Groenendaal F Van Den Bekerom MPJ Doornberg JN Van Haarst EP Riezebos R. Predicting peripheral venous catheter related phlebitis using machine learning (PPML): development and prospective validation of PPML for cardiology inpatients. BMC Med Inform Decis Mak. (2025) 25(1):316. 10.1186/s12911-025-03158-6 40866970 PMC12382011

[17] Manrique-Rodríguez S Heras-Hidalgo I Pernia-López MS Herranz-Alonso A Del Río Pisabarro MC Suárez-Mier MB. Standardization and chemical characterization of intravenous therapy in adult patients: a step further in medication safety. Drugs R D. (2021) 21(1):39–64. 10.1007/s40268-020-00329-w PMC7937591 33346878

[18] Ballesteros-Peña S Fernández-Aedo I Vallejo-De la Hoz G Tønnesen J Miguelez C. Identification of potentially irritating intravenous medications. Enferm Intensiva (Engl Ed). (2022) 33(3):132–40. 10.1016/j.enfie.2021.05.003 35941074

[19] Ferraz-Torres M Moreno-Rubio E Cobo-Sánchez JL Alarcón-Duque EM Suárez-Mier MB Río-Pisabarro MDCD. Pharmacological burden and chemical phlebitis associated with peripheral venous catheters: a nested case-control study. Results of the national flebitis zero project. Heart Lung. (2026) 79:102836. 10.1016/j.hrtlng.2026.102836 42160916

[20] Gorski LA Ong J Van Gerpen R Nickel B Kokotis K Hadaway L. Development of an evidence-based list of non-antineoplastic vesicants: 2024 update. J Infus Nurs. (2024) 47(5):290–323. 10.1097/NAN.0000000000000568 39250767

[21] Soeters PB Wolfe RR Shenkin A. Hypoalbuminemia: pathogenesis and clinical significance. JPEN J Parenter Enteral Nutr. (2019) 43(2):181–93. 10.1002/jpen.1451 30288759 PMC7379941

[22] Valeriani E Pannunzio A Palumbo IM Bartimoccia S Cammisotto V Castellani V. Risk of venous thromboembolism and arterial events in patients with hypoalbuminemia: a comprehensive meta-analysis of more than 2 million patients. J Thromb Haemost. (2024) 22(10):2823–33. 10.1016/j.jtha.2024.06.018 38971499

[23] Furlan MDS Saba A Lima AFC. Risk factors associated with the occurrence of the adverse event phlebitis in hospitalized adult patients. Rev Bras Enferm. (2024) 77(5):e20240162. 10.1590/0034-7167-2024-0162 39383435 PMC11458144

[24] Privitera D Geraneo A Li Veli G Parravicini G Mazzone A Rossini M. Complications related to short peripheral intravenous catheters in patients with acute stroke: a prospective, observational, single-cohort study. Intern Emerg Med. (2024) 19(6):1605–13. 10.1007/s11739-024-03651-2 38805082 PMC11405487

[25] Blanco-Mavillard I Personat-Labrador C Castro-Sánchez E Rodríguez-Calero MÁ Fernández-Fernández I Carr PJ. Interventions to reduce peripheral intravenous catheter failure: an international e-delphi consensus on relevance and feasibility of implementation. J Infect Public Health. (2023) 16(12):1994–2000. 10.1016/j.jiph.2023.10.004 37890222

[26] Xu L Hu Y Huang X Fu J Zhang J. Clinically indicated replacement versus routine replacement of peripheral venous catheters in adults: a nonblinded, cluster-randomized trial in China. Int J Nurs Pract. (2017) 23(6):e12595. 10.1111/ijn.12595 28990241

[27] Kang N Kim HL Choi HR Kim J. Routine versus clinically indicated replacement of peripheral intravenous catheters in adults: a non-inferiority cluster-randomised crossover trial. Healthcare (Basel). (2026) 14(13):1825. 10.3390/healthcare14131825 42450834 PMC13361898

[28] Rickard CM Marsh N Webster J Runnegar N Larsen E McGrail MR. Dressings and securements for the prevention of peripheral intravenous catheter failure in adults (SAVE): a pragmatic, randomised controlled, superiority trial. Lancet. (2018) 392(10145):419–30. 10.1016/S0140-6736(18)31380-1 30057103

[29] Ray-Barruel G Xu H Marsh N Cooke M Rickard CM. Effectiveness of insertion and maintenance bundles in preventing peripheral intravenous catheter-related complications and bloodstream infection in hospital patients: a systematic review. Infect Dis Health. (2019) 24(3):152–68. 10.1016/j.idh.2019.03.001 31005606

[30] Laan BJ Nieuwkerk PT Geerlings SE. Patients knowledge and experience with urinary and peripheral intravenous catheters. World J Urol. (2020) 38(1):57–62. 10.1007/s00345-018-02623-4 30680418 PMC6954151

[31] Doll MS Aprile DCB Gonçalves ALP Da Silva BSM Kusahara DM Lopes CT. Development and content validity of a questionnaire on peripheral intravenous catheter maintenance and knowledge of nursing professionals regarding best practices. J Infus Nurs. (2025) 48(1):53–69. 10.1097/NAN.0000000000000571 39760879

