# Supplementary material for: Development and external validation of a risk prediction model for early peripheral intravenous catheter failure in outpatients and emergency department patients receiving consecutive infusion therapy
Source: Front Med (Lausanne). 2026 Oct 8;13:1963710. doi: 10.3389/fmed.2026.1963710 (PMC13687379; doi:10.3389/fmed.2026.1963710)
Supplement: Supplementary file 2 [file Table1.docx]

| Category | Operational definition | Representative medications in the present study |
| --- | --- | --- |
| pH-related irritant medications | Intravenous medications whose pH after preparation or under the actual infusion conditions deviated substantially from the physiological range and for which prescribing information, pharmaceutical references, or previous studies indicated potential local venous irritation. | Intravenous azithromycin; levofloxacin injection; aminophylline injection; pantoprazole for injection; omeprazole for injection |
| Hyperosmolar medications | Intravenous medications that, under the actual clinical preparation and infusion regimen, imposed a relatively high osmotic load and could potentially increase peripheral venous irritation. | 20% mannitol injection; potassium chloride injection; calcium gluconate injection; calcium chloride injection; high-dose intravenous vitamin C |
| Particulate/excipient-related irritant medications | Intravenous medications that may increase local venous irritation because of complex formulation characteristics, excipients or emulsifying systems, or recognized risks of particulate formation, aggregation, or precipitation during preparation or administration. | Compound multivitamin preparation for injection; Xuesaitong for injection; salvianolate for injection; Shenmai injection; fat-soluble vitamin preparation for injection |

Supplementary Table S1. Operational Definitions and Representative Medications of Potentially Irritant Intravenous Medication Categories
